# Development and Characterization of Nanostructured Pharmacosomal Mesophases: An Innovative Delivery System for Bioactive Peptides

**DOI:** 10.15171/apb.2018.069

**Published:** 2018-11-29

**Authors:** Maryam Rezvani, Javad Hesari, Seyed Hadi Peighambardoust, Maria Manconi, Hamed Hamishehkar

**Affiliations:** ^1^Department of Food Science, College of Agriculture, University of Tabriz, Tabriz, Iran.; ^2^Department of Environmental and Life Science, University of Cagliari, Cagliari, Italy.; ^3^Drug Applied Research Center, Tabriz University of Medical Sciences, Tabriz, Iran.

**Keywords:** Bioactive peptide, Hypertension, Liquid crystal, Lyotropic, Nanoparticle, Sustained release

## Abstract

***Purpose:*** To potentially enhance the bioavailability and extend the bioactivity effectiveness of Isoleucine-Proline-Proline (IPP, an antihypertensive bioactive peptide of dairy origin), a novel Lyotropic Liquid Crystalline Pharmacosomal Nanoparticle (LLCPNP) was synthesized, and its physicochemical and technological characteristics were studied.

***Methods:*** LLCPNPs precursors were developed using IPP and soy phosphatidylcholine via complex formation. Polarized light microscopy, small angle X-ray scattering, differential scanning calorimetry, dynamic light scattering and Fourier transform infrared spectroscopy were employed to characterize the physicochemical properties of the nanoparticles. The in-vitro release and its related mechanisms were also studied.

***Results:*** Fourier transform infrared spectroscopy confirmed the complexation between the components of LLCPNPs. Phase behavior evaluation by polarized light microscope showed the characteristic birefringent texture. These findings along with those of small angle X-ray scattering and differential scanning calorimetry proved the formation of lamellar LLCPNPs. These particles represented nanometric size (<100 nm), high incorporation efficiency (93.72%) and proper physicochemical stability during long-term storage. In-vitro studies demonstrated a sustained release behavior fitted to non-Fickian diffusion and Higuchi kinetic models.

***Conclusion:*** The present study results emphasized that LLCPNPs could be proposed as an unrivaled carrier to promote the bioavailability, stability and shelf-life of nutraceutical and biopharmaceutical formulations containing bioactive peptides.

## Introduction

**Figure 1:**
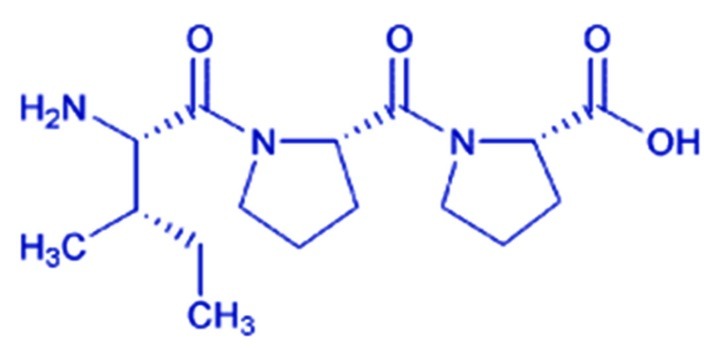
Chemical structure of Isoleucine-Proline-Proline.

In recent decades, application of the Lyotropic Liquid Crystals (LLCs) in the field of drug delivery has gained considerable attraction from many scientists and research communities.^1-6^ The unique and peculiar physicochemical and structural characteristics of LLCs, similarity to the bio-systems and providing superior advantages than traditional delivery systems are the causes of this scientific interest. These structures are considered as an intermediate state of matter between a crystalline solid (consisting the long-range positional and orientational order in three dimensions) and an isotropic liquid (with no long-range order), which need a solvent to be formed. This state may simultaneously represent the crystal mechanical stability and liquid fluidity.^7-9^ These mesophases have been utilized to protect and deliver some molecules with different biological activities such as immunosuppressive and anticancer agents, antibiotics and vitamins.^10-12^ Regarding the importance of bioactive peptides in biopharmaceutical applications, the employment of LLCs should be considered as a noteworthy method for delivery of these valuable compounds. Isoleucine-Proline-Proline (IPP) is an antihypertensive bioactive peptide with dairy origin. Hypertension is the most effective risk factor causing cardiovascular disease (CVD), which has become an important global public health concern and will change to the leading cause of death in subsequent years. In addition, high blood pressure is the cause of many other Illnesses, including kidney failure, stroke and premature death.^13^ Angiotensin I-converting enzyme (ACE) plays a key role in this regard. This enzyme can raise blood pressure through catalyzing the conversion of angiotensin I to angiotensin II and vasoconstriction.^14^ Many of the antihypertensive drugs inhibit this process, but these synthetic ACE inhibitors have numerous side effects. In recent years, some peptides with food origin have shown ACE inhibitory properties. The utilization of these bioactive peptides reduces the cost of hypertension treatment and adverse side effects.^15-18^ In this regard, the function of IPP has been prominent, but its undesirable pharmacokinetics, including low bioavailability, short half-life, rapid clearance from the body and probable interaction with the other components in oral delivery formulations necessitate the need for nanoencapsulation of this bioactive peptide.^19^ On the other hand, the chemical structure of this molecule makes it appropriate to produce specific LLCs, Lyotropic Liquid Crystalline Pharmacosomal Nanoparticles (LLCPNPs), via complex formation (Figure 1).

The present study aims to investigate the physicochemical characteristics of LLCPNPs, for potential application in oral delivery formulations containing bioactive peptides with extended antihypertensive therapeutic effects. To achieve this objective and due to the reasons mentioned above, IPP was used as a model peptide. The physicochemical characterization of LLCPNPs was performed by means of small angle X-ray scattering, Fourier transform infrared spectroscopy, differential scanning calorimetry, polarized light microscopy and dynamic light scattering. Furthermore, *in-vitro* release behavior and storage stability were studied. The combination of these methods allows elevation of knowledge about these novel structures and elucidation of their applications in peptide delivery.

## Materials and Methods

### Materials

Isoleucine-Proline-Proline (IPP) was purchased discounted from Bachem AG, Bubendorf, Switzerland. Soy phosphatidylcholine (SPC) was provided from LIPOID GmbH, Ludwigshafen, Germany. Milli-Q water was directly produced using Millipore Corporation system (Burlington, MA, USA). All the other chemicals were of analytical grade.

### Preparation of liquid crystalline structures

To synthesize LLCPNPs precursor, IPP was allowed to react with equilmolar concentration of SPC. The reaction was accomplished at 25 °C for 4 hours in a sealed atmosphere chamber under gentle agitation at constant speed in an ethanolic medium. Subsequently, LLCPNPs precursor was deposited by a rotovap, exposed to nitrogen flushing and collected as dried residues. A predetermined amount of the precursor was hydrated at a neutral pH, homogenized (SilentCrusher M, Heidolph, Burladingen, Germany) at 20000 rpm and sonicated by a probe sonicator (Hielscher, Teltow, Germany) at 70% amplitude for 5 cycles (1 min ON and 1 min OFF) in an ice bath to form the uniform lyotropic liquid crystalline nanostructures. For comparison, the nanoparticles without IPP (ENPs) were prepared in the same manner. The resultant structures were lyophilized (LyoQuest, Telstar, Spain) for 24 h and kept in amber-colored vials at low temperature.

### Characterization of optimized LLCPNPs

### Fourier Transform Infrared (FTIR) spectroscopy

A FTIR spectrometer (Tensor 27, Bruker, Ettlingen, Germany) using KBr pellet technique was employed to characterize the SPC, IPP and LLCPNPs precursor.

### Differential Scanning Calorimetry (DSC)

Thermal behavior studies of LLCPNPs, ENPs and IPP were recorded by a differential scanning calorimeter (Mettler-Toledo, Gießen, Germany). All the scans were performed in a nitrogen gas atmosphere over a temperature range of -50 °C to 200 °C with a constant heating rate of 5 °C/min.

### Polarized Light Microscopy (PLM)

A polarized light microscope (MPL-1, BEL, milan, Italy) was used as a preliminary method to distinguish between anisotropic and isotropic systems and characterize the phase of fully hydrated LLCPNPs precursor at room temperature.

### Small Angle X-ray Scattering (SAXS)

SAXS patterns of LLCPNPs were acquired by X-ray scattering apparatus (S3-MICROpix, Hecus, Graz, Austria) with a one-dimensional sensitive position detector (1D-SPD) at room temperature with Cu Kα radiation (λ= 1.542 Å). The scattering factor equation (1), and Bragg equation (2) are calculated as follows.^20^

q=4πsinθ/λ     (1)

2dsinθ=nλ     (2)

In these equations, q and θ are the scattering factor and angle of the incident ray, respectively. λ Is the wavelength of X-ray beam and d is the distance between the lattice layers. A new equation (3) is obtained from the combination of equations 1 and 2:

d=2π/q     (3)

In this work, the scattering patterns were obtained by plotting the scattering intensities as a function of the scattering factor.

### Determination of incorporation efficiency 

The amount of IPP actual incorporated into the LLCPNP structure was assessed by a centrifugation method employing an Amicon® Ultra-15 tube (Millipore, Darmstadt, Germany) at 5000 rpm for 20 min and determination of free IPP concentration in the lower chamber of Amicon® tube. A novel rapid isocratic HPLC (Knauer, Berlin, Germany) method with C18 column (5 µm particle size, 4.6 mm I.D. × 150 mm L) was used to determine the amount of IPP in the solutions with UVmax absorbance at 215 nm. A mixture of acetonitrile: phosphate buffer (pH 2.85) with the ratio of 75:25 (v/v) at the flow rate of 1 ml/min was selected for sample elution. Phosphate buffer was prepared by dissolving 34 mg of potassium dihydrogen phosphate in 90 ml of Milli-Q water, adjusting the pH to 2.85 with phosphoric acid and diluting to 100 ml with milli-Q water.^21^ The mobile phase was exposed to the vacuum filtration and degassing ultrasonication to protect the column. The linearity of calibration curve was evaluated in different concentrations of IPP. The Incorporation efficiency (IE) was calculated according to the following equation:

IE%=(Mass of IPP incorporated into LLCPNP/Mass of total IPP)×100     (4)

### Particle size distribution and zeta potential

The hydrodynamic diameter, polydispersity index (PDI) and zeta potential of the liquid crystalline nanoparticles were determined by dynamic light scattering (DLS) technology (Zetasizer Nano ZSP, Malvern, Worcestershire, UK) after dilution of a suitable amount of fresh LLCPNPs dispersion at neutral pH and 25 °C.

### Stability studies 

The stability of the optimized formulation of LLCPNPs was quantitatively assessed during 30 days storing at 4 °C. For this purpose, sampling was carried out for different time intervals and IE values, mean hydrodynamic diameter and PDI were measured as described in the previous sections.

### Release studies

*In-vitro* release profiles were obtained using a dynamic dialysis technique by sealing of LLCPNPs dispersion (2 ml, 3 mg/ml) in a dialysis bag and incubating at 37±0.5 °C in phosphate buffer solution (pH 7.4) with total volume of 200 ml. The stock buffer solution was prepared by mixing potassium dihydrogen phosphate (0.2 M, 250 mL) and sodium hydroxide (0.1 M, 393.4 mL).^21,22^ The release media solution was continuously stirred at 100 rpm. At predetermined time intervals, 2 ml of this solution were withdrawn and replaced with the equal volume of fresh media to maintain sink conditions. The filtered withdrawn solutions were analyzed for IPP content by HPLC as described previously. The release behavior of the free IPP in the release medium was assayed for comparison.

### Release kinetics

The mechanism of the IPP release was determined by fitting the* in-vitro* release data to different mathematical models i.e. Zero order, First order, Higuchi, Weibull and Hixson–Crowell kinetic models.^23^ Moreover, the data were fitted to Ritger-Peppas equation to confirm the release mechanism.^24^ The goodness-of-fit test based on coefficient of determination (R-squared) was the criterion for selecting the most appropriate model.

### Statistical analysis

Analysis of the experimental data was conducted using Duncan’s mean comparison test at the significant level of 5% and one-way ANOVA with SPSS software (Version 19.0). Measurements were conducted in triplicate and the data were expressed as mean± standard deviation (SD).

## Results and Discussion

### FTIR spectroscopy

**Figure 2:**
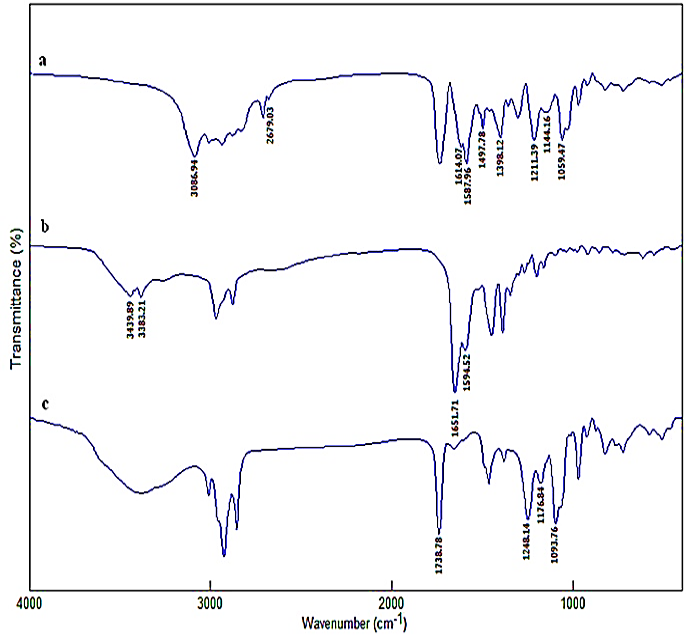
Fourier transform infrared spectra of nanoparticles precursor (a), Isoleucine-Proline-Proline (b) and soy phosphatidylcholine (c)

To substantiate the complex formation in LLCPNPs precursor, its infrared spectrum and the spectra of its components were evaluated, which showed the significant changes in the former compared to the latters (Figure 2). In the IPP spectrum, two bands at 3439.89 cm^-1^ and 3383.21 cm^-1^ referred to –NH_2_ stretching, overlapping with the wide OH stretching vibration extended from 3500 cm^-1^ to 2500 cm^-1^ due to intramolecular hydrogen bonding. The peaks at 1651.71 cm^-1^ and 1594.52 cm^-1^ indicated the C=O and N–H bending. The stretching at 1738.78 cm^-1^referred to the presence of esteric carbonyl group in the SPC spectrum. The vibration peaks corresponding to P=O, P–O and P–O–C appeared at 1248.14, 1176.84 and 1093.76 cm^-1^, respectively. In the complex spectrum, the OH stretching vibration of IPP disappeared and two new peaks appeared at 1398.12 and 1587.96 cm^-1^, representing the characteristic absorption (symmetric/asymmetric) of –COO-, covering the umbrella like vibration of –NH_3_^+^. The wavenumbers relevant to the vibration peaks of phosphate group diminished. The stretching vibrations of –NH_2_ disappeared and the broad peak appeared between 3086.94 cm^–1^ and 2679.03 cm^–1^, including the overlapping of the stretching bands related to –NH3^+^ and C–H. Moreover, the characteristic asymmetric and symmetric N–H bending vibrations of –NH_3_^+^ appeared at 1614.07 and 1497.78 cm^-1^. All these observations confirmed the establishment of the interactions between IPP and SPC and complex formation. These findings were compatible with the results obtained in previous studies.^22,25-29^

### DSC analysis 

**Figure 3:**
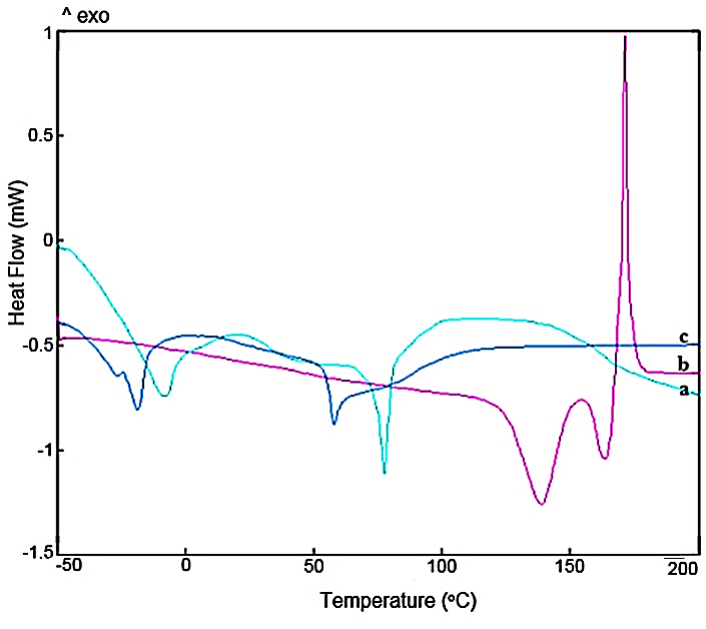
Differential scanning calorimetry thermograms of empty nanoparticles (a), Isoleucine-Proline-Proline (b) and lyotropic liquid crystalline pharmacosomal nanoparticles (c)

In the DSC technique, appearance, elimination or change in enthalpy, heat content and position of the endothermic or exothermic peaks can be considered as an interaction occurrence.^30^ in this work, the thermogram of IPP showed an endothermic peak at 138.92 °C followed by an endothermic/exothermic peak probably due to its degradation (Figure 3). The characteristic peaks of IPP disappeared in the DSC thermogram of LLCPNPs. The properties of the complex peaks differed from those of ENPs and IPP. These findings confirmed the results of the other experiments such as FTIR and proved the complex formation. Furthermore, these results emphasized that the complexation led to establish a less ordering lipidic structure. The resulted structures were in the liquid crystalline state in the conditions under which the experiments were conducted. These findings are in accordance with those acquired by Maryana, et al.^31^ and Semalty et al.^32^

### PLM evaluation

One of the most important techniques to identify various types of mesophases is microscopic investigation based on their different optical activities under polarized light, employed as a preliminary phase screening method for SAXS measurements. The liquid crystalline structures showed birefringence by PLM. However, the cubic LLCs cannot be detected using this property because of their isotropic behavior.^33-35^

**Figure 4:**
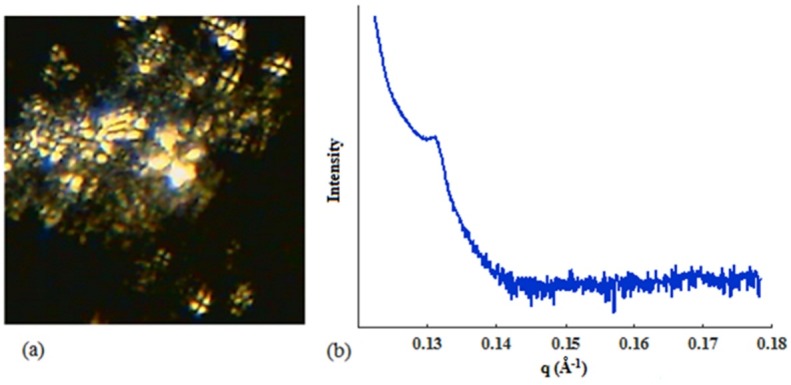
Polarized light micrographs (a) and small angle X-ray scattering pattern (b) of the lyotropic liquid crystalline pharmacosomal nanoparticles

Figure 4a shows the micrograph of LLCPNPs under polarized light microscope. The Maltese cross pattern formation in the presence of polarized light is the fundamental property of L_α_ liquid crystals. The appearance of this pattern is different from the fan-shaped texture, which is typical for lyotropic hexagonal and thermotropic smectic liquid crystals.^28,34^ These characteristics represent the lamellar liquid crystalline nature of LLCPNPs.

### SAXS assessments

In this study, SAXS measurements were performed to understand the inner structure of the LLCPNPs. X-ray technique not only makes it possible to evaluate the structure of the LLC phases but also specifies the presence of long-range order.^36^ The scattering patterns of the samples were plotted measuring the intensity of scattered waves as a function of scattering angle. Strong intensities, known as Bragg peaks, were obtained at the points where the scattering angles satisfy the Bragg conditions (Figure 4b). The scattering pattern can be considered as a fingerprint of a structure. The different distances in LLCPNPs can be obtained by locating the Bragg peaks. A relatively broad reflection peak at q of 0.131 Å^-1^ disclosed a long-range ordered structure, being highly consistent with the results of PLM. All these observations indicate the lamellarity of LLCPNPs. According to the Bragg’s law, the lamellar repeat spacing (d) in LLCPNPs studied in this research is larger than that of the samples with no complexation. This phenomenon confirms the important impact of the complex formation on the internal structure of these mesophases.

### Particle size distribution and zeta potential 

**Figure 5:**
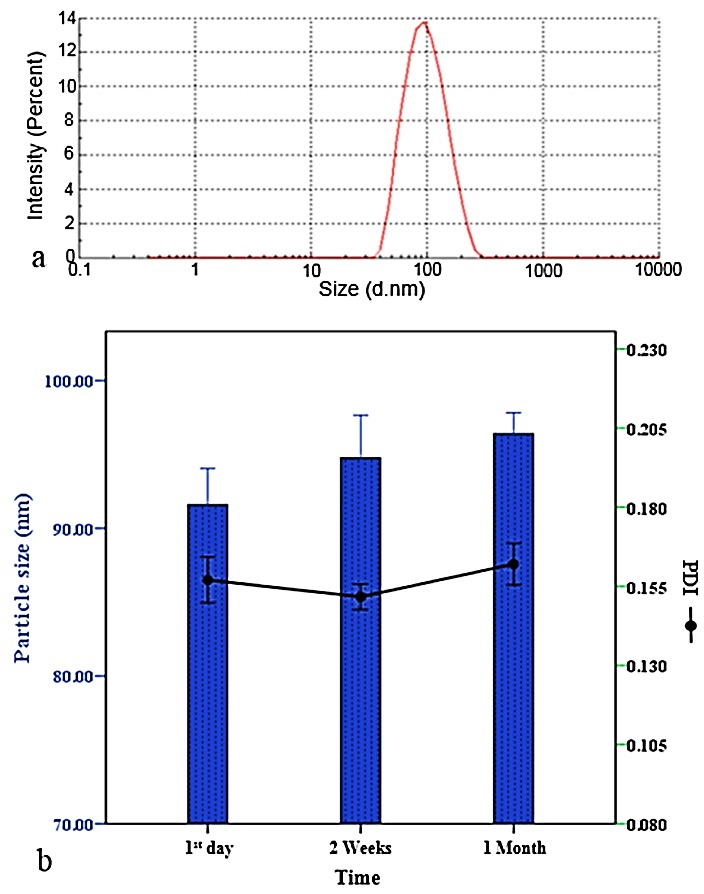
Particle size distribution of the fresh lyotropic liquid crystalline pharmacosomal nanoparticles (a) and changes in particle size and polydispersity index (PDI) during 30 days storing at 4 °C (b). Results are expressed as mean ± SD (n=3). No significant difference was found between the mean values (P>0.05).

The stability and interactions between the particles in the suspensions were evaluated by measuring the zeta potential, being an indicator of charge distribution on the surfaces of the particles. Moreover, the size of the particles is another valuable factor in evaluation of physicochemical stability, *in-vivo* absorption and bioavailability of delivery systems. The penetration rate across the gastrointestinal barrier is estimated to be approximately 250 times higher for nanoparticles than microparticles.^37^ On the other hand, macrophages exhibit the highest levels of phagocytosis on particles with sizes between 150 nm and 2 μm, while nanoparticles with the size range of 70 to 150 nm prolong the plasma concentration of delivered molecule.^38^ The measured mean hydrodynamic diameter (Figure 5a), PDI and zeta potential of LLCPNPs were 91.6±1.98 nm, 0.157±0.01 and -24.1±0.63 mV, respectively. These obtained values indicated that the designed nanostructures were sufficiently uniform, small and stable to act as a successful system for effective delivery of the bioactive peptides, resulting in bioavailability improvement.

### Stability studies 

The results of stability studies on optimized LLCPNPs showed no significant changes in the IE value after 30 days, demonstrating no degradation in nanostructure during the storage period. Based on the DLS results, the size remained in nano-range (<100 nm) during the storage period (Figure 5b), and no significant changes were observed in mean hydrodynamic diameter and PDI of LLCPNPs. This observation could be attributed to relatively small size and appropriate zeta potential of the nanostructures providing strong electrostatic repulsion and physical stability over the time.

### In-vitro release studies

**Figure 6:**
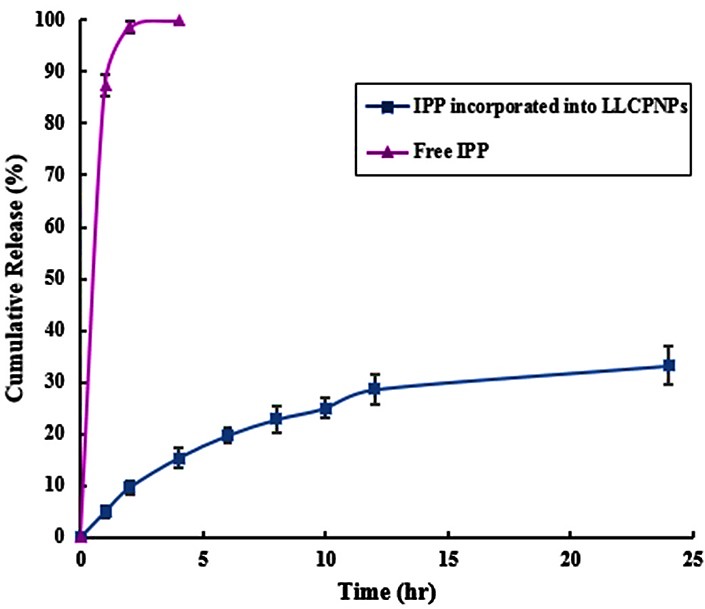
*In-vitro* release profiles of free Isoleucine-Proline-Proline and incorporated into nanoparticles. Data are expressed as mean±SD (n=3).

The employment of* in-vitro* release studies of drug delivery systems to predict their *in-vivo* behavior and reach the goal of controlled release has been very noteworthy. In the present work, *in-vitro* release studies were conducted in pH 7.4 to simulate the blood conditions. The release profile of IPP incorporated into LLCPNPs was obtained and compared to that of free IPP (Figure 6). As this figure presents, t_50_ (time required for 50% release of peptide) of free IPP is much less than that of IPP incorporated into LLCPNP formulations indicating a retarded release of IPP from LLC structures. This phenomenon could be interpreted by considering the changes in the state of dynamic equilibrium between monomer and complexed forms of IPP, LLCPNPs and their disassociated forms and IPP concentration inside and outside the dialysis bag. All these equilibrium equations together probably determined the release rate of IPP from LLCs to the simulated acceptor solution.

### Release kinetics 

The most appropriate model to evaluate the IPP release mechanism was selected based on the coefficient of determination and goodness-of-fit test on corresponding *in-vitro* release data. The peptide release patterns for IPP incorporated into LLCPNPs followed weibull kinetics (Table 1).

Better interpretation and characterization of the controlled release mechanism were provided by evaluating the release exponent (n) calculated by Ritger–Peppas equation, which confirmed an anomalous (non-Fickian) mechanism for this release study.

**Table 1:** The R-squared (R^2^) values and equations for* in-vitro* release of Isoleucine-Proline-Proline from lyotropic liquid crystalline pharmacosomal nanoparticles fitted to different release models

**Model**	**Equation**	**R** ^ 2 ^	**n**
**Zero order**	Q=7.7445+1.3382t	0.7983	-
**First order**	ln100-Q=4.5257-0.0166t	0.8382	-
**Higuchi**	Q = - 0.0095+ 7.5148t^1/2^	0.9666	-
**Hixon-Crowell**	Q^1/3^=4.5127-0.0238t	0.8252	-
**Weibull**	log⁡(-ln1-Q)=0.4891+0.0084logt	0.9787	-
**Rittger-Peppas**	lnQ = 0.7751+ 0.6109lnt	0.9586	0.6109

**n:** release exponent,** Q:** amount of drug released at time t, **t:** time

## Conclusion

The successful preparation technique and appropriate physicochemical characteristics of LLCPNPs containing IPP acquired in this work can be applicable for the similar compounds. According to the results of the SAXS and PLM, these nanoparticles can be classified as lamellar LLC phases. As illustrated in this research, the utilization of this nanocarrier type in peptide delivery can increase the patient tolerance and decrease the dosage and administration frequency of the peptide via the steady plasma concentration maintenance at the desired therapeutic level for a longer period. The peptide release from these novel LLCs is independent of the carrier membrane fluidity, leading to sustained release of IPP. This feature along with the bioavailability promotion can result in the promising potential for manufacturing the biopharmaceutical and nutraceutical products aiming at preventing and controlling the diseases originated from hypertension and can improve the health.

## Ethical Issues

The present paper does not contain any studies involving animals or human participants.

## Conﬂict of Interest

There are no conflicts to declare.
